# MEASURING SPACE AND TIME USE, PHYSICAL AND SOCIAL ACTIVITIES AMONG NON-ENGLISH SPEAKING LATE-LIFE ASIAN IMMIGRANTS

**DOI:** 10.1093/geroni/igz038.709

**Published:** 2019-11-08

**Authors:** Wenjun Li, Linda Churchill, Jie Cheng, Rachel Siden, Annabella Aguirre, Kevin Kane

**Affiliations:** University of Massachusetts Medical School, Worcester, Massachusetts, United States

## Abstract

Little is known about the health and health care needs of non-English Speaking late-life Asian immigrants. Due to language barriers and memory issues, self-report data are unreliable for investigating activity patterns in this population. In the ongoing NIA-funded Healthy Aging and Neighborhood Study, we developed a novel method to objectively measure space and time use, location- and time-specific physical and social activities using accelerometer (ACC) and Global Positioning System (GPS) devices. The study has recruited over 150 Caucasians and 150 minorities including 50 non-English speaking late-life Asian immigrants. The participants answered surveys in their preferred language (English, Spanish, traditional or simplified Chinese) and wore ACC/GPS devices for 7 to 10 full days. Activity levels and geographic locations are recorded every 30 seconds. Using the combined ACC/GPS data, time- and location-specific activity amounts, time use and mobility patterns are objectively measured. Baseline findings will be reported at the GSA conference.

